# The Functional Role of PRC2 in Early T-cell Precursor Acute Lymphoblastic Leukemia (ETP-ALL) – Mechanisms and Opportunities

**DOI:** 10.3389/fped.2016.00049

**Published:** 2016-05-18

**Authors:** Kathrin M. Bernt, Stephen P. Hunger, Tobias Neff

**Affiliations:** ^1^Department of Pediatrics, Center for Cancer and Blood Disorders, Children’s Hospital Colorado, University of Colorado Denver, Aurora, CO, USA; ^2^Department of Pediatrics, Center for Childhood Cancer Research, The Children’s Hospital of Philadelphia, Philadelphia, PA, USA

**Keywords:** leukemia, lymphoid, epigenetics, polycomb repressive complex, Hox genes, JAK/STAT signaling pathway

## Abstract

Early T-Cell precursor acute lymphoblastic leukemia (ETP-ALL) is a relatively newly identified subset of T-lineage ALL. There are conflicting results regarding prognosis, and the genetic basis of this condition is variable. Here, we summarize the current status of the field and discuss the role of mutations in the Polycomb Repressive Complex 2 frequently identified in ETP-ALL patients.

## ETP-ALL, A Novel Subtype of Acute Lymphoblastic Leukemia

In 2009, Coustan-Smith and colleagues reported a subtype of T-lineage acute lymphoblastic leukemia (T-ALL) with transcriptional and surface marker profile similarities to early T-cell precursors (ETP-ALL) (1). Clinical characteristics of ETP-ALL include associations with lower WBC, older age, and a very high rate of poor MRD response or even induction failure at the end of induction chemotherapy.

Initial reports suggested that ETP-ALL has an extraordinarily poor long-term prognosis, with 2- to 10-year event free/overall survival (EFS/OS) rates in the 11–40% range, compared to 84–90% OS for all pediatric T-ALL in a similar time frame (1–3). However, patients in these cohorts were not treated on the most recent treatment protocols [e.g., 1992–2006 for the St. Jude cohort and 2001–2006 for the AIEOP cohort (1)] and included only a small number of ETP-ALL patients: 17 in the St Jude, 13 patients in the AEIOP (1), 5 in the Tokyo Children’s Cancer Study Group L99-15 cohort (2), 12 in the Shanghai Children’s (3), and 7 (3 pediatric and 4 adult) treated at Columbia University (4). Poor outcomes were also reported for adult patients with immune-phenotypic ETP-ALL or T-ALL with a gene expression profile characteristic of ETP-ALL (5–7).

## ETP-ALL in Relation to Other Definitions of Immature T-ALL

Early T-cell precursor acute lymphoblastic leukemia is defined based on immunophenotyping (CD1a-negative, CD8-negative, weak CD5 expression with less than 75% positive blasts, and expression of one or more of the following myeloid or stem cell markers on at least 25% of lymphoblasts: CD117, CD34, HLA-DR, CD13, CD33, CD11b, and/or CD65). It is noteworthy that other definitions of very immature T-ALL subtypes have been proposed. In an important study, using array-based genome-wide expression profiling, Ferrando et al. found that T-ALL with *LYL1* expression clusters separate from other T-ALL subtypes and show immunophenotypically immature features (8). The Meijerink-group identified an immature subgroup of T-ALL with high *MEF2C* expression, which based on expression profiling clustered separately from other T-ALL cases and was enriched for ETP-ALL cases (9). This same laboratory went on to further characterize the overlap between ETP-ALL defined by gene expression profile, ETP-ALL based on immunophenotyping, MEF2C-high T-ALL, and a third group of immature T-ALL originally described by Gutierrez et al. defined by absence of biallelic TCRgamma deletion (ABD) (10). Poor prognosis for T-ALL and T-lymphoblastic lymphoma with ABD was also found in two other studies (11, 12). Meijerink and colleagues systematically compared the different definitions of immature T-lineage ALL. There is excellent overlap between the immature cluster and ETP-ALL as defined by an ETP-ALL gene expression signature (13). There is less overlap between the immature cluster and ETP-ALL defined by immunophenotyping. Inclusion of the CD5 marker in flow-cytometry panel reduced the number of cases classified as phenotypic ETP-ALL. Omission of the CD5 marker improved the number of expression-based ETP-ALL cases that also were immunophenotypically defined as ETP-ALL. However, the less stringent flow criteria also led to the classification as immunophenotypic ETP-ALL of samples without an ETP-ALL expression profile. There is also imperfect overlap between samples with ABD and immature as well as transcriptionally defined ETP-ALL.

In summary, there are four potential classification methods for early/undifferentiated T-lineage ALL. (1) Immature expression profile cluster, (2) ETP-ALL expression profile, (3) ETP-ALL immunophenotype, and (4) ABD. A MEF2C-high immature cluster seems to mostly overlap with transcriptionally defined ETP-ALL, and a case has been made that this is one biological entity (13). The ETP-ALL phenotype depends on complex criteria, and single features such as the CD5 strongly influence classification, limiting sensitivity and specificity of the flow panel. ABD seems to only partially overlap with the other subgroups based on the data reported in Ref. (10, 13). The overlap between the different diagnostic samples in Ref. (13) is shown in Table 1.

**Table 1:** **Overlap between different methods to classify immature T-lineage ALL [data from Ref. (13)]**.

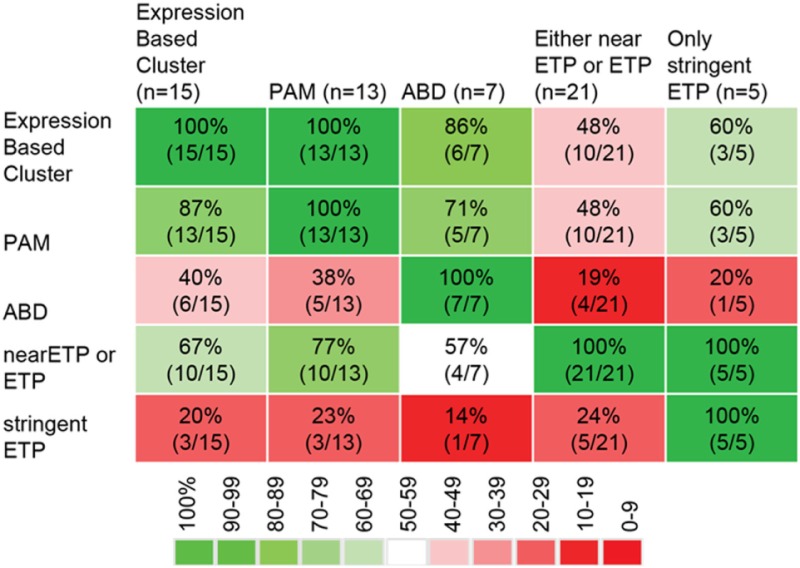

*There is excellent overlap between clustering and PAM based on a defined ETP-ALL signature. Stringent ETP-ALL criteria including CD5 low criterion exclude many samples classified as immature by gene expression profile. Relaxing the CD5 criterion (“near ETP”) includes a number of samples that do not display an ETP-ALL expression profile. PAM, prediction analysis of microarrays*.

## Improved Outcomes in ETP-ALL in More Recent Studies

More recent studies from Europe and the US demonstrated a substantially better survival for patients with immature T-lineage ALL, compared to the reports referenced above. The Dutch group reported non-inferior survival of immature ALL in their study using all four definitions outlined above (13). Patrick et al. recently reported results for patients treated in England and Wales. In this series of patients, treated on protocol UKALL 2003 between 2003 and 2011, results for patients with phenotypic ETP-ALL were only slightly and non-significantly worse than results for cases with typical T-ALL phenotype (14). To address the outcome of different T-ALL subsets with contemporary therapy, the Children’s Oncology Group (COG) used a single central reference laboratory to classify 1144 T-ALL cases enrolled on the COG AALL0434 clinical trial between 2007 and 2014 as ETP [methodology harmonized with the approach used by Coustan-Smith et al. (1)], near ETP (ETP-ALL flow profile but positive CD5 staining), and not ETP. The five-year EFS/OS for 130 (11.3%) ETP-ALL cases was 87.0/93.0%, compared to 84.4/91.6% for 195 (17.0%) near-ETP-ALL cases, and 86.9/92.0% for 819 (71.6%) not-ETP cases, showing that ETP-ALL is not associated with an inferior outcome with contemporary treatment regimens (15).

There are several possible reasons for these discrepant outcomes. One obvious reason is that the flow-cytometric definition of this subtype of ALL is not straightforward. In theory, differences of how this patient population is defined could account for differences in outcome. For example, 69% of patients in the UKALL 2003 ETP-ALL cohort, which reported good outcomes for ETP-ALL, were classified as “probable ETP-ALL” (with CD5 staining either positive or unavailable). Only 31% were classified as “definitive ETP-ALL,” again speaking to the difficulty of accurately defining this patient population. Outcomes showed a non-statistically significant trend toward lower EFS (76.7 vs. 84.6%) and OS (82.4 vs. 90.9%) for the group of ETP-ALL (“probable” + “definitive”), but the absolute number of definitive ETP-ALL patients was small (11 patients) and outcomes for this group were not reported separately. However, it should be noted again that the recent US data were classified by the reference laboratory that initially reported ETP-ALL. In the Dutch study, outcomes were non-inferior to typical T-ALL using gene expression-based and immune-phenotypic classification criteria as well as ABD criteria.

A second possible reason may be that more recent treatment regimens are in fact superior to older regimens. An important aspect of more recent protocols is the implementation of MRD-based, risk-adapted treatment strategies. Analysis of clinical and outcomes characteristics of ETP-ALL patients treated on the COG ALL0434 and UKALL 2003 showed that very few ETP-ALL patients fell into the low-MRD group, similar to what has been observed by other investigators. Importantly, MRD response at end consolidation on newer trials is not clearly different from MRD response observed in other types of T-ALL, suggesting that some of the poor therapeutic response observed in ETP-ALL may be related to the specific drugs used during induction chemotherapy. It is tempting to speculate that resistance to corticosteroids in particular may be important in the poor response of ETP-ALL to induction chemotherapy. Yet, early identification of these patients based on intermediate or high MRD, and increased intensity of therapy may have offset the higher-risk biology of ETP-ALL, resulting in the good outcomes in these two trials. In addition, differences in choice of agents may influence results. As pointed out by Zuurbier et al., COALL-97 includes high-dose Cytarabine (13). A subset of patients on COG ALL0434 was randomized to receive Nelarabine. The precise impact of including these drugs on outcome is presently not known. The outcomes for the different cohorts are summarized in Table 2.

**Table 2:** **Survival data from published studies in ETP-ALL and ABD-ALL**.

Reference	Cohort	Year	Age (years)	EFS (%)	OS (%)	years follow up	Classification
ETP-ALL (pediatric, initial reports)							
(1)	US/SJCRH	2009	0.5–18.9	19	22	10	FC
(1)	Italy/AEIOP		^b^	45	22	2	FC
(2)	Japan/Tokyo Children’s Cancer Study Group L99-15	2012	1–18	40	~70	4	FC^a^
(3)	China/Shanghai Children’s Medical Center	2012	^c^	11	13	3	FC
(4)	US/Columbia University	2013	7–49	14	86	5	FC
ABD							
(10)	US/COG, DFCI	2010	2–17	25	25		PCR
(11)	Taiwan/TPOG	2012	^c^	43	46	10	PCR
(12)	France/EuroLB02	2012	<20	0	0	3	PCR
ETP-ALL (pediatric, recent reports)							
(13)	Holland/DCOG	2014	1.5–17.8	70	^c^	5	multiple
(15)	US/COG	2014	^c^	87	93	5	FC
(14)	UK/UKALL2003	2014	1.0–24.9	77	82	5	FC
Adult data							
(5)	Germany/GMALL	2012	^d^	^c^	35	10	FC
(6)	Europe/E2993 ECOG	2013	^d^	^c^	34	5	GEP
(7)	US/MDACC	2016	13–79	^f^	^g^	5	FC
(46)	France/GRAALL	2016	^e^	25	31	5	FC

*The study by Callens et al. describes four cases of lymphoblastic lymphoma with ABD*.

*ABD, absence of biallelic deletion of the gamma/delta T-cell receptor; FC, flow cytometry; GEP, Gene expression profiling*.

*^a^Custom score involving flow cytometry*.

*^b^62% 1–9 years, 38% >10 years*.

*^c^Not available*.

*^d^Adult patients, exact age range not available*.

*^e^Median age 30.5*.

*^f^Median OS = 17 months*.

*^g^Median EFS = 20 months*.

## Genetic Heterogeneity and Outcome

A third potential reason lies in the genetic heterogeneity of ETP-ALL. The seminal study by Zhang et al. (16) identified a very high rate of mutations in certain functional “clusters” of genes. These include signaling-associated genes (particularly *Il7R, NRAS, KRAS, FLT3, JAK1, JAK3*, and *SH2B3*), developmental genes (including genes encoding the transcription factors *GATA3*, *RUNX1*, *ETV6*, and *IKZF1*), and epigenetic modifiers [including inactivating alterations in components of the Polycomb Repressive Complex 2 (PRC2)] (16). The frequencies for alterations individual components among ETP-ALL were as follows: *SUZ12* 11/64 cases, *EED* 8/64 cases, *EZH2* 10/64 cases, any PRC2 alteration 26/64 cases. Of note, three out of the ETP-ALL patients had lesions in two PRC2 member. Fifteen out of a total of 29 PRC2 alterations in ETP-ALL were deletions, strongly suggesting they are inactivating. Similarly, 6 out of 6 PRC2-alterations found in 42 cases of typical T-ALL were deletions. While the *EZH2* mutations found were not all individually functionally characterized in this study, based on computer modeling they are thought to be inactivating (16). This is in contrast to the activating mutations of *EZH2* described in B-lineage lymphomas (17, 18). The discovery (whole genome sequencing) cohort in this study consisted of a limited number of 12 patients, with targeted sequencing only in the larger validation cohort. A larger cohort allowing for a more complete definition of the genomic landscape of ETP-ALL would be very informative. In multi-variant analysis, mutations in PRC2 members were found to be an independent predictor of poor outcome. It is possible that outcomes differences between ETP-ALL patient cohorts could read out differences in the underlying genomic landscape. The mutational landscape for the patients in the study by Wood et al. (15) is currently being investigated (19). Preliminary analyses of patients in the UKALL2003 cohort suggest ETP-ALL patients in this cohort showed some of the genetic characteristics of ETP-ALL (less frequent *NOTCH1/FBXW7* mutations, no *LMO1/2* or *TLX1* mutations, and increased rate of *KMT2A* translocations). However, alterations of PRC2 components were not reported for this cohort. It is also noteworthy that in adult ETP-ALL, in contrast to pediatric cases, recurrent *DNMT3A* alterations were found in 16% (20) and 7.5% (6). This is in keeping with low proportions of *DNMT3A* mutations in pediatric acute leukemia (21, 22) and with high proportion of *DNMT3A* alterations in age-related clonal hematopoiesis of indeterminate potential (23).

## A Murine Model to Study the Role of PRC2 in ETP-ALL

The association between survival and PRC2 alterations in the study by Zhang et al. (16) was noteworthy. PRC2 is a multiprotein complex with important roles in cancer and development. PRC2 consists of the core components EZH2, EED, and SUZ12 (24). PRC2 di- and tri-methylates histone 3 on lysine 27, a genetic modification associated with low or no transcriptional output. Importantly, high-level expression of *EZH2* has been linked to poor outcomes in solid tumors, but recent reports have cast doubt on H3K27me3 as the culprit (25, 26). PRC2-independent roles for *EZH2* have been reported, illustrating the complexity of PRC2 biology (25, 27, 28). Wild-type *EZH2* and naturally occurring hyperactive mutants have been found to be important in the development of Germinal center B-lineage lymphomas, and EZH2 is a promising target for small molecule inhibition in this disease (17, 18). An epistatic relationship between PRC2 and the SWI/SNF chromatin remodeling complex (also known as BAF complex) has been reported (29, 30). Given that approximately 20% of human cancer has alterations in SWI/SNF (31, 32), there is growing interest in EZH2 as a therapeutic target (29, 30, 32).

Despite these bona-fide oncogene credentials, recurrent inactivating alterations of PRC2 genes have been described in ETP-ALL (16) and other hematologic and non-hematologic malignancies (33–41), creating somewhat of a paradox: PRC2 can have oncogenic and tumor-suppressive roles in cancer depending on cellular context. How precisely EZH2/PRC2 exert their oncogenic and tumor suppressor roles mechanistically is incompletely understood. A better understanding of the molecular details would likely aid in the development of targeted therapies.

**Figure 1:**
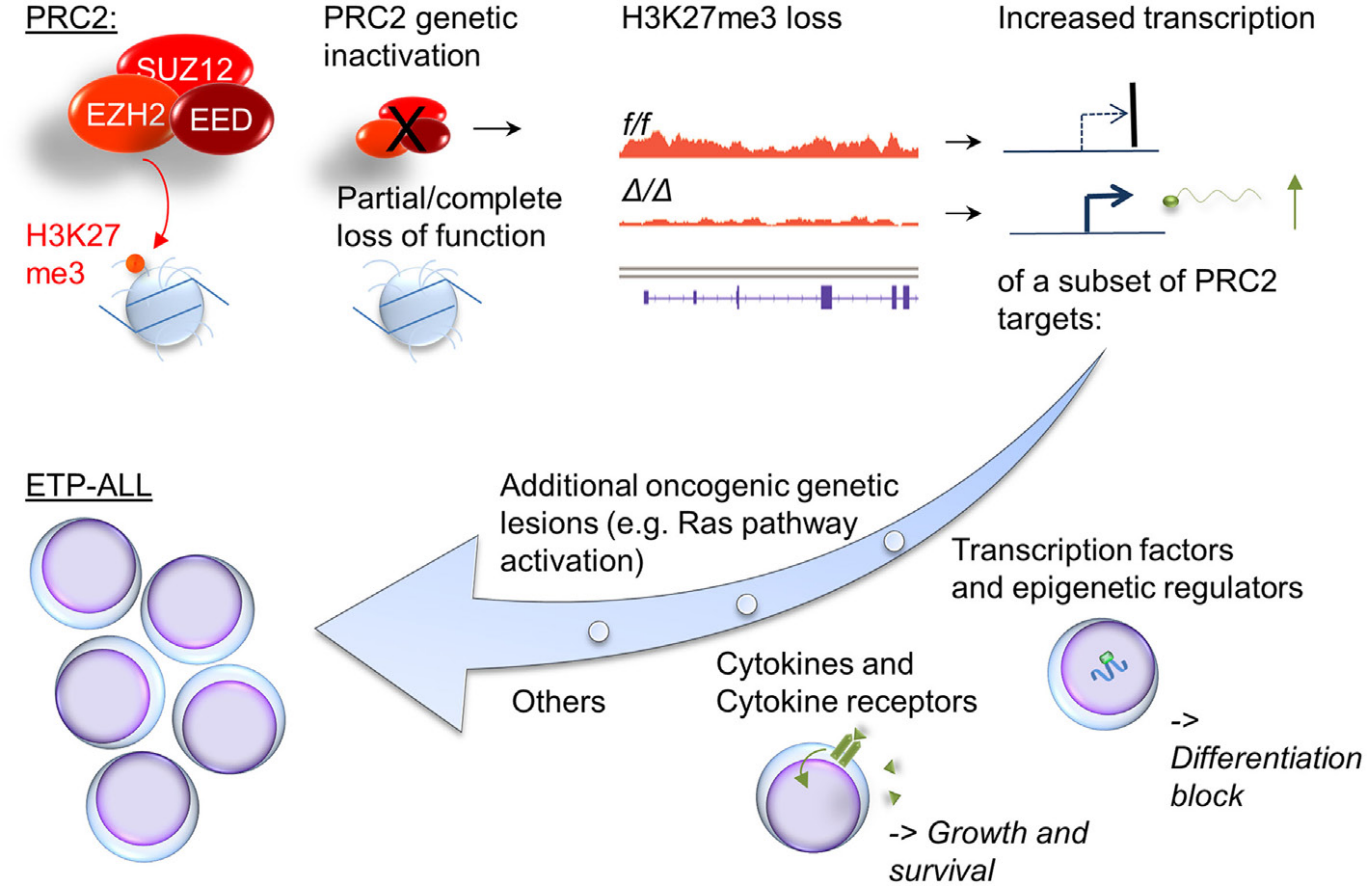
**Cellular consequences of compromised PRC2 function in ETP-ALL**. Inactivation of PRC2 components in ETP-ALL results in cellular loss of global cellular H3K27me3, a chromatin mark associated with silent genes. A subset of these genes show increased transcription. Noteworthy groups of target genes with increased expression in Ras-transformed cells with compromised PRC2 function include transcription factors and epigenetic regulators associated with early hematopoiesis (e.g., *HoxA*, *Gata2*, and *Bmi1*) and growth factors and their receptors (e.g., *Il6ra*).

To clarify the functional role of PRC2 in T-ALL, we recently generated a murine model of ETP-ALL that allows for the comparison of leukemias with and without genetic compromise in PRC2 function (42). Since RAS-activation occurs frequently in ETP-ALL, we overexpressed oncogenic mutant *NRAS* in immature hematopoietic cells. We used mice with homozygously floxed alleles for the PRC2 components *Eed* and *Ezh2*, allowing homozygous inactivation of either PRC2-member. The mice also contained homozygously deleted *Cdkn2a* alleles, to facilitate leukemogenesis [genetic Cdkn2a inactivation is very common in T-ALL, is not typical for human ETP-ALL (43), but is required for successful establishment of the model]. Cells were then differentiated toward a T-cell fate on stromal cells providing a Notch-signal, and injected into lethally irradiated mice. We found that 100% of mice developed leukemia/lymphoma that recapitulated some features of human ETP-ALL (e.g., lack of CD4 and CD8 expression in most samples, low expression of CD5, and co-expression of myeloid markers). Inactivation of *Ezh2* or *Eed* in this model significantly shortened leukemia latency. The findings from our model are summarized in Figure 1. We noted that *Ezh2* inactivation is associated with the expression of a stem cell gene expression program. This includes *HoxA9*, and genes correlated with *HOXA9* in human leukemia. Interestingly, three recent reports suggest a prognostic role for *HOXA9* in ETP-ALL: a subset of T-ALL patients enrolled in AALL00434 was analyzed, and the authors concluded that patients with the combination of MLL-rearrangement, high *HOXA9* expression, and ETP-ALL (by expression profiling) had a significantly worse outcome (44). Similarly, T-ALL patients with the *CALM-AF10* fusion, which is associated with high *HOXA* expression (45), had a worse prognosis if blasts showed an immature phenotype (46). In a French cohort, the combination of high *HOXA* expression and ETP-ALL immunophenotype showed significantly inferior outcomes. In this study, a number of the high *HOXA* expressors were explained by chromosomal translocations, but many were not. The PRC2 mutational status in this study was not investigated (47). These data provide a good rationale for paying special attention to the combination of ETP-ALL and HOXA expression in future studies.

Our murine study also identified deregulation of growth and survival signaling as a direct consequence of loss or PRC2 components. Similarly to what was reported by De Raedt et al. and others in NF1-associated sarcomas (36), we found that in a malignancy with activated RAS signaling, a RAS-downstream signature is further enriched by compromise in PRC2 function. In addition, we found deregulated expression of cytokines and their receptors in response to *Ezh2*-inactivation. *Il6ra* is a known PRC2 silencing target, and its derepression in our model was accompanied by exaggerated STAT3Y705 phosphorylation in response to IL6. The clinically approved JAK2 inhibitor ruxolitinib inhibited growth in our model *in vitro*. Importantly, David Teachy’s group recently demonstrated pre-clinical efficacy of ruxolitinib in human ETP-ALL samples (48). Our data suggest that even samples without obvious activating mutations in the JAK/STAT signaling pathway may respond to JAK/STAT small molecule inhibitors. This was indeed the case for two patient samples without JAK/STAT pathway mutation, but with an inactivating PRC2 component mutation, in the xeongraft study by Maude et al (48). The incorporation of JAK-inhibitors in ETP-ALL should be evaluated in more detail in pre-clinical trials and possibly also in carefully designed clinical studies, with a thoughtful selection of patients most likely to benefit.

## Additional Genetic Lesions in ETP-ALL

It is noteworthy that certain genetic lesions are inversely correlated with PRC2 alterations. These include mutations in *GATA3*, which also carry a poor prognosis (16). It is tempting to speculate that GATA3 and PRC2 collaborate in T-cell development, and that their inactivation in ETP-ALL has some degree of functional redundancy. Furthermore, it is interesting to note that PRC2 alterations and *FLT3* alterations are mutually exclusive in the study by Zhang et al. (16). More experimental work and larger studies that interrogate these suggestive molecular findings and correlate molecular alterations with survival data seem warranted.

Based on the data available today, alterations in treatment are not warranted for children with an ETP-ALL immunophenotype detected at the time of initial diagnosis (and data in adults may be too sparse to draw definitive conclusions). However, there is a higher rate of poor early response, and overt induction failure in ETP-ALL, and most relapses that occur in ETP-ALL occur early in therapy. Thus, there is a subset of ETP-ALL patient (and/or those with an immature immunophenotype) who relapse quickly with extremely refractory disease, or never go into remission in the first place. This raises the following pressing questions for the field:

1. Is flow cytometry plus early treatment response the best strategy to identify high-risk patients, or is there a less equivocal marker (ETP-transcriptional signature? PRC2 mutation? Kinase signature?) that can identify the group of patients with ETP-ALL and a poor outcome?
2. Assuming that ultra-high-risk patients can be identified at diagnosis or very early in therapy can advances in understanding the biology of the disease pave the way toward targeted and more effective therapies?

## A Broader Role for Polycomb Genes in Tumor Biology?

The combination of PRC2 inactivation and oncogenic RAS pathway activation now has been observed in several contexts: NF1-associated malignant peripheral nerve sheath tumors (MPNST) (36–38), juvenile myelomonocytic leukemia (JMML) (39, 40), megakaryocytic AML of Down syndrome (41), and ETP-ALL (16). In addition, mutations in genes other than *EED*, *SUZ12*, and *EZH2* with a documented effect on canonical PRC2 function are inactivated in cancer (including hematologic neoplasms). This includes, e.g., Jarid2 (49–51) and Asxl1 (52). Finally, genes involved in Polycomb Repressive Complex 1 have shown tumor suppressor function in model systems (53, 54). In summary, these findings suggest commonalities between the roles of different Polycomb genes in cancer that may potentially be targetable. As an example, the chromatin-binding protein BRD4 has been suggested as such a therapeutic target (36). The careful study of patient material and genetic models holds great promise in this regard. We are optimistic that a deeper understanding of the molecular mechanisms underlying this collaboration will result in successful future clinical trials and ultimately in improved outcomes for patients.

## Author Contributions

All authors listed, have made substantial, direct, and intellectual contribution to the work and approved it for publication.

## Conflict of Interest Statement

The authors declare that the research was conducted in the absence of any commercial or financial relationships that could be construed as a potential conflict of interest.
